# Editorial: Host immune response and regulation to parasitic infections: therapeutic approaches and defence strategies

**DOI:** 10.3389/fimmu.2023.1215086

**Published:** 2023-05-24

**Authors:** Arif Jamal Siddiqui, Mohd Adnan, Fevzi Bardakci, Adebayo James Molehin

**Affiliations:** ^1^ Department of Biology, College of Science, University of Ha’il, Ha’il, Saudi Arabia; ^2^ Department of Microbiology and Immunology, College of Graduate Studies, Midwestern University, Glendale, AZ, United States

**Keywords:** parasites, immune response, vaccine immunology, drug development, natural killer cell, macrophage, dendritic cell, T and B cells

Parasites are responsible for causing serious illnesses in humans. Drugs and vaccines are the most effective tools for providing significant health benefits, saving millions of human lives, and decreasing disease burden and death. Advanced immunological technologies for vaccine development have emerged in the last decade to fight parasitic and numerous other severe diseases. To date, however, licensed vaccines that protect against parasitic diseases are not commercially available, except for malaria. Although drug resistance in most parasites and the absence of vaccines seem to be extremely dangerous in terms of the threat to human life, advancements in immunological research and tools continue to improve our knowledge regarding drug and vaccine development (1, 2). This understanding is significant for clinical applications and has facilitated the discovery of novel drugs, vaccines, diagnostics, and treatments to manage a wide array of parasitic diseases such as malaria, schistosomiasis, filariasis, leishmaniasis, and trypanosomiasis.

In this Research Topic, a total of eight articles were published, covering recent advancements in parasitic diseases. The first article summarizes the identified plasma cytokine pathways that can cause disease in individuals susceptible to *Leishmania guyanensis* infection. While a common response to leishmaniasis is observed, this research suggests that IP-10 (interferon gamma-induced protein 10), IL-2 (interleukin-2), and RANTES (regulated on activation, normal T expressed and secreted) may have predictive values for disease development, while PDGF (platelet-derived growth factor), IL-1Ra, and eotaxin may be protective indicators. In addition, IL-17 and IL-1 are potential indicators of disease progression in individuals infected by *Leishmania guyanensis.* Finding possible targets along these signaling pathways could facilitate the development of immunotherapy (Mesquita et al., 2022).

The second article highlights one of the most important parasitic infections in the freshwater fish Myxobolus: a particularly deadly species of the genus Myxospora. Liu et al. conclude that immunity is inhibited, while p53-Bcl2/Bax-associated networks dominating the expression of apoptotic genes are activated in the gills of goldfish infected with *Myxobolus ampullicapsulatus*. This article is useful for improving the taxonomy of Myxobolus and elucidating its pathogenic mechanism, and offers targets for myxobolosis control and prevention.

The third article examines the dynamic m^6^A (N6-Methyladenosine) mRNA methylation patterns in mice infected by *Plasmodium yoelii*. This article demonstrates the importance of m^6^A as a post-transcriptional regulatory mechanism in malaria parasite infections. By controlling m^6^A-modifying enzymes, infection with malaria parasites significantly alters the m^6^A mRNA modification profile and host gene expression in the spleen. Describing the precise contributions and signaling mechanisms of key molecules that control the host m^6^A methylome during parasitic malaria infection will be important for future studies and may aid in the development of vaccines or drugs to treat malaria (Wang et al., 2022).

The fourth article presents the role of protein glycosylation in protective immunity against a parasitic worm and fully describes important implications for the development of vaccines against metazoan parasites. Wang et al. provide the first experimental evidence that N-glycosylation of the H11 glycoprotein complex in *Haemonchus contortus* intestinal microvilli is essential for immune protection.

The fifth article uses the EgPSC-infected (Protoscoleces *Echinococcus granulosus*) mouse model to mimic an *Echinococcus granulosus* reinfection scenario by injecting *Echinococcus granulosus* Protoscoleces intraperitoneally. For the first time, Zhou et al. report that non-oral transmission of EgPSC infection results in gut injury and immune metabolism reprogramming in mice, and that Mebendazole administration alleviates these changes, indicating that unimpaired gut barrier function is crucial for protection against secondary *E. granulosus* infection.

The sixth article presents the effects of biosynthesized selenium nanoparticles against *Eimeria papillata* –induced infections in C57BL/6 male mice. Abdel-Gaber et al. show that these Bio-SeNPs have anticoccidial activity and histological changes are significantly improved in the mouse jejunum.

The seventh article demonstrates that intestinal epithelial cells (IECs) express a ROS-AMPK/mTOR-mediated autophagy mechanism in response to *Giardia* infection. Furthermore, *Giardia*-induced downregulation of the IEC-TJ (tight junction) protein and reduction in nitric oxide release, rather than autophagic flux, are associated with the regulatory role of early-stage autophagy (Wu et al., 2023).

The eighth and final article demonstrates that GRA5 (granule proteins) is essential for pathogenicity and cyst development of *Toxoplasma gondii*. The intratumoral injection of ME49Δgra5 shows excellent efficacy against metastases in the lungs as well as against the growth of injected and removed 4T1 tumors. ME49Δgra5 injection boosts splenic innate, adaptive immune, and tumor infiltrating cells, and also promotes IFN-γ and IL12 production. ME49Δgra5 is identified as a potential immunotherapeutic for malignancy and a promising vaccine against *T. gondii* infection (Chen et al., 2023).

These contributions and thorough mechanistic investigations on the developmental progress of various parasite vaccines, parasite biology, parasite immunology, and the development of new parasite drugs provide various approaches for the treatment of parasitic infections. Nevertheless, parasites remain a serious public health problem worldwide, requiring continuous research into new therapeutic agents, vaccine development, and treatment approaches. The guest editors would like to extend their sincere thanks to all authors for their significant contributions.

## Author contributions

Conceptualization and edited this special Research Topic, AS, MA, FB, and AM. All authors contributed to the article and approved the submitted version.
